# The Anti-Inflammatory Properties of Polysaccharides Extracted from *Moringa oleifera* Leaves on IEC6 Cells Stimulated with Lipopolysaccharide In Vitro

**DOI:** 10.3390/ani14233508

**Published:** 2024-12-04

**Authors:** Hosameldeen Mohamed Husien, Weilong Peng, Mohamed Osman Abdalrahem Essa, Saber Y. Adam, Shahab Ur Rehman, Rahmat Ali, Ahmed A. Saleh, Mengzhi Wang, Jingui Li

**Affiliations:** 1 College of Veterinary Medicine, Yangzhou University, Yangzhou 225009, China; 008643@yzu.edu.cn (H.M.H.); wlpeng98@163.com (W.P.); mohosman0999@gmail.com (M.O.A.E.); 2 College of Veterinary Medicine, Albutana University, Rufaa 22217, Sudan; 3 Laboratory of Metabolic Manipulation of Herbivorous Animal Nutrition, College of Animal Science and Technology, Yangzhou University, Yangzhou 225009, China; shahabgul99@gmail.com (S.U.R.); rahmatalihu@gmail.com (R.A.); 4 College of Animal Science and Technology, Yangzhou University, Yangzhou 225009, China; saaber5757@gmail.com (S.Y.A.); elemlak1339@gmail.com (A.A.S.); 5 Animal and Fish Production Department, Faculty of Agriculture (Al-Shatby), Alexandria University, Alexandria City 11865, Egypt; 6 State Key-Laboratory of Sheep Genetic Improvement and Healthy-Production, Xinjiang Academy of Agricultural Reclamation Sciences, Shihezi 832000, China

**Keywords:** *M. oleifera*, intestinal epithelial cells, signalling pathways, inflammatory response, gut barrier

## Abstract

*Moringa oleifera* (*M. oleifera*) is a plant with significant medicinal and nutritional value and contains various bioactive compounds, particularly in its leaves (MOL). This study sought to explore the impact of *M. oleifera* leaf polysaccharides (MOLPs) on lipopolysaccharide (LPS)-activated intestinal epithelial cells (IEC6) and to uncover the mechanisms involved. The cytotoxicity of MOLP on IEC6 cells was assessed using the Cell Counting Kit-8 (CCK-8) assay, which demonstrated a safe concentration range of 0–1280 µg/mL. The impact of MOLP on cell viability was further evaluated over 12 to 48 h. IEC6 cells were treated with three concentrations of MOLP low (25 µg/mL), medium (50 µg/mL), and high (100 µg/mL) alongside LPS (50 µg/mL) stimulation for one day. The findings revealed that treatment with MOLP significantly promoted cell migration and increased the production of interleukin-10 (*IL-10*), while it simultaneously decreased cell apoptosis and the levels of pro-inflammatory cytokines, such as tumour necrosis factor alpha (*TNF-α*), interleukin 1β (*IL-1β*), and interleukin 6 (*IL-6*). Additionally, MOLP treatments across all concentrations significantly reduced the expression of Toll-like receptor 4 (TLR-4), myeloid differentiation primary response 88 (MyD88), phosphorylated nuclear factor kappa B-alpha (pIκB-α), and phosphorylated NF-κB p65 signalling pathways. Moreover, MOLP restored the expression of tight junction proteins, such as zonula occludens-1 (ZO-1) and occludin, which had been disrupted by LPS. These results indicate that MOLP exhibits anti-inflammatory properties by inhibiting inflammatory signalling pathways and maintaining intestinal barrier integrity through the upregulation of tight junction proteins in IEC6 cells. This study enhances our understanding of the anti-inflammatory capabilities of MOLP.

## Simple Summary

Intestinal health is crucial for overall well-being. Polysaccharides extracted from *Moringa oleifera* leaves (MOLP) may offer significant anti-inflammatory benefits for intestinal tissues. This study examines how MOLP affects intestinal epithelial cells (IEC6) activated by lipopolysaccharide (LPS) in a lab setting, focusing on inflammation markers and key signalling pathways. The results show that MOLP enhances cell migration, reduces cell death, and decreases inflammatory cytokines, while also restoring the proteins that maintain intestinal barrier integrity. These findings suggest that MOLP can help manage inflammation and support intestinal health.

## 1. Introduction

The intestinal epithelium is shielded by a single layer of intestinal epithelial cells (IECs), which play crucial roles in digesting food, absorbing nutrients, and defending the body against harmful substances, allergens, and infections [1,2]. This epithelial layer is exposed to more physical stressors than other body tissues, which requires maintaining homeostasis [3]. When the intestinal lining sustains an injury, neighbouring IECs move to the affected region and divide to preserve the integrity of the intestinal mucosa [4]. However, disorders like inflammatory bowel disease (IBD) lead to recurrent harm to the mucosal surface of the intestines, causing impairment in IEC function [5,6,7]. Therefore, promoting IEC migration and proliferation could be an effective therapeutic strategy for intestinal diseases.

*Moringa oleifera* (*M. oleifera*) (commonly known as the “drumstick tree”) is a versatile perennial tree found in Southeast Asia, Africa, southern China, and other parts of the world [8,9]. Almost every part of *M. oleifera* offers significant biological and nutritional benefits [10,11]. The leaves (MOL) are particularly valuable, being rich in phenols, glucosinolates, proteins, fats, and essential minerals. The leaves are reported to exhibit bioactivities such as anti-inflammatory and antioxidant properties [12,13,14]. In addition, the seeds, flowers, and gum of *M. oleifera* contain nitrile glycosides (niazirin and niazirinin), flavonoid, and thiocarbamate glycosides, which have anti-inflammatory, antibacterial, anti-diabetic, and anti-hypertensive properties [15,16,17,18]. In vitro research has demonstrated that various components of *M. oleifera* exhibit numerous physiological and pharmacological benefits, attributed to the numerous bioactive compounds they harbour [19,20,21].

Inflammation serves as the body’s natural defence mechanism against injuries caused by both non-living and living factors, such as bacterial infections and irritants [22,23]. Cytokines such as tumour necrosis factor (*TNF-α*) and interleukin 6 (*IL-6*) are included in the regulation of inflammation and the body’s defence mechanisms. While unchecked and severe inflammation can lead to immune disorders, immune cell apoptosis can result in cancer and chronic degenerative diseases [24,25,26]. Thus, managing inflammation appropriately is crucial, and involves the balanced production of pro-inflammatory mediators and cytokines [27].

Research has shown that *Panax genus* polysaccharides possess anti-tumour and immunomodulatory properties [28]. Similarly, sulphated galactofucan from *Lobophora variegata* exhibits anti-inflammatory effects [29], and polysaccharides derived from jujube (*ziziphus-jujuba* Mill) demonstrate significant protective effects against colorectal cancer induced by azoxymethane (AOM)/dextran sulphate sodium (DSS) [30]. However, the potential impact of MOLP on inflammation in vitro, especially in lipopolysaccharide (LPS)-induced IEC6 cells, and the mechanisms included are not yet completely understood. Exploring the potential of *Moringa oleifera* leaf polysaccharides (MOLP) reveals significant insights into innovative treatments for intestinal disorders characterised by tissue damage. Mohamed et al. [31] reported that these polysaccharides demonstrate a multifaceted approach to health by exerting anti-inflammatory and antioxidant effects, crucial in mitigating conditions like ulcerative colitis. MOLP supports the restoration and maintenance of intestinal barrier integrity by enhancing the expression of tight junction proteins such as zonula occludens-1 (ZO-1) and occludin, essential for preventing intestinal permeability. Moreover, MOLP modulates inflammatory responses by down-regulating pro-inflammatory signalling pathways, including the Toll-like receptor 4 (TLR4) and nuclear factor-kappa B (NF-κB) pathways, while up-regulating anti-inflammatory mediators like interleukin-10 (*IL-10*) and peroxisome proliferator-activated receptor-γ (PPAR-γ). These actions not only prevent the infiltration of inflammatory cells but also maintain mucosal structure, highlighting MOLP as a promising therapeutic approach for addressing inflammatory conditions and enhancing overall intestinal health.

In this aspect, inflammation is a key component of many intestinal diseases, often leading to severe health issues if not properly managed. *M. oleifera*, widely known for its nutritional and medicinal benefits, contains polysaccharides that have shown potential anti-inflammatory properties. Understanding the specific effects of these polysaccharides on intestinal cells can help develop new therapeutic approaches to manage and possibly mitigate inflammatory conditions in the gut. This study will provide crucial insights into *M. oleifera* potential role in reducing inflammation and enhancing intestinal health, offering a natural approach to address this widespread problem [22,31].

The main objectives of this study were to investigate the anti-inflammatory effects of polysaccharides derived from *Moringa oleifera* leaves on IEC6 cells stimulated with LPS and to unravel the mechanisms behind these effects. Specifically, the research aimed to examine how MOLP influences cell migration, viability, and apoptosis, as well as its impact on cytokine production and inflammatory signalling pathways.

## 2. Materials and Method

### 2.1. Plant Source and Extraction of Polysaccharide from M. oleifera

In accordance with our prior research [31], *M. oleifera* leaf (MOL) powder was sourced from Yunnan-Ruziniu Biotechnology, Kunming, China. Subsequently, polysaccharides were extracted from the MOL powder, referred to as MOLP, following the extraction procedures outlined in earlier studies [31,32,33].

### 2.2. Structural Analysis of MOLP

In a previous study, we detailed the characteristic properties, molecular weight, and monosaccharide composition of MOLP [31].

### 2.3. IEC-6 Cell Culture

The IEC6 cells were cultivated in Dulbecco’s Modified Eagle Medium (DMEM) medium supplemented with 10% fetal bovine serum (FBS). The cells were seeded into 25 cm^2^ culture flasks, maintained at 25 °C, and incubated in a constant-temperature setting of 37 °C with 5% CO_2_ and saturated humidity. An inverted microscope was used to monitor cell proliferation. Once the cells reached over 90% confluence in the culture flask, the medium was discarded, and the cells were rinsed twice with phosphate-buffered saline (PBS). Subsequently, 1 mL of 0.25% trypsin was added for enzymatic digestion, which lasted approximately 3 min. When microscopic observation confirmed cell detachment, 2 mL of complete DMEM was rapidly added to halt the digestion. The cells were then collected and centrifuged (1100 rpm/7 min). Following centrifugation, the cells were re-suspended in a fresh medium and transferred into a new-culture flask at a 1:3 dilution ratio. All subsequent experimental procedures were conducted after the cells had been passaged three times.

### 2.4. Cell Viability

The viability of IEC6 cells treated with MOLP was defined utilising the methodologies described in previous research [34]. The cells were exposed to different concentrations of MOLP low (25 µg/mL), medium (50 µg/mL), and high (100 µg/mL) for 48 h. Following the treatment period, the culture medium was discarded, and the wells were rinsed twice with PBS. Fresh DMEM (100 µL per well) containing Cell Counting Kit-8 (CCK-8) (10 µL per well) was subsequently added. A microplate reader was used to detect the absorbance at 490 nm following 2 h of incubation at 37 °C. Cell viability was determined with the following formula: cell viability (%) = (A2/A1) × 100%, where A1 represents the absorbance of the control sample and A2 represents the absorbance of the treated sample. To ensure reproducibility, each experiment was conducted in triplicate.

### 2.5. Cell Migration

Cell motility was assessed following the methodologies described in previous studies [34,35]. In brief, IEC6 cells were grown in 6-well culture plates containing DMEM (Nanjing, China) supplemented with 10% FBS (Nanjing, China). Uniform scratches were made using sterile 200 µL pipette tips once cellular confluence was achieved. The cells were then washed three times with PBS and imaged utilising a phase contrast microscope at 100× magnification (0 h). After a 1 h incubation in serum-free media (SFM) (Nanjing, China), the cells were treated with either MOLP-L (25 µg/mL), MOLP-M (50 µg/mL), MOLP-H (100 µg/mL), or LPS (100 µg/mL) for 48 h. The scratches were re-photographed under the same conditions at 24 and 48 h post-treatment, after another set of three PBS washes. The distances travelled by the cells were quantitatively analysed using ImageJ software (Image J1.53).

### 2.6. Cell Apoptosis

The apoptosis rate of IEC6 cells was evaluated using flow cytometry. Following 24 h of treatment with MOLP-L (25 µg/mL), MOLP-M (50 µg/mL), and MOLP-H (100 µg/mL) in combination with LPS (100 µg/mL), the cells were harvested and stained with fluorescein isothiocyanate (FITC)-conjugated Annexin V and propidium iodide (PI) (Nanjing, China) for 20 min at 25~27 °C in the absence of light. The apoptotic rate was then quantified utilising flow J analysis software (Image J1.53), as outlined by Li et al. [36].

### 2.7. RT-qPCR Analysis

IEC6 cells were seeded into 6-well plates at a density of 4 × 10^5^ cells per well and incubated for one day. Subsequently, they were exposed to MOLP-L (25 µg/mL), -M (50 µg/mL), -H (100 µg/mL), or LPS (50 μg/mL) for an additional day. The next steps were mentioned in our previous research [31]. The specific primers used for the target genes are listed in Table 1.

### 2.8. Western Blot Approach

IEC-6 cells were cultured in 6-well plates (4 × 10^5^ cells/well) and incubated for 24 h, and then stimulated with MOLP-L (25 µg/mL), MOLP-M (50 µg/mL), MOLP-H (100 µg/mL), or LPS (50 μg/mL) for 24 h, respectively. After incubation, the cells were harvested, collected, and lysed in a radioimmunoprecipitation assay (RIPA) buffer containing a phenylmethylsulfonyl fluoride (PMSF) protease inhibitor while kept on ice. The cell lysates were then centrifuged (12,000 rpm/10 min) at 4 °C. As described in our previous study [31], a bicinchoninic acid (BCA) protein assay kit was used to determine the protein concentration of the supernatant. After being boiled with loading buffer, equal amounts of protein in each sample were prepared for electrophoresis on 10% sodium dodecyl sulphate–polyacrylamide gel (SDS-PAGE) under the reducing conditions, transferred to immobilon-p polyvinylidene fluoride (PVDF) membrane, and blocked with 5% skimmed milk at room temperature for 2 h, followed by incubation with specific primary antibodies at 4 °C overnight (all antibodies were diluted following instructions). After washing with Tris-buffered saline Tween (TBST) three times, samples were incubated with species-specific horseradish peroxidase-conjugated secondary antibodies at room temperature for 1 h. After washing with TBST three times, protein signal bands were visualised on a Chemidoc XRS (BIO-RAD, Marnes-la-Coquette, France) using an enhanced chemiluminescence kit (Merck Millipore, Billerica, MA, USA) and quantified using ImageJ software.

### 2.9. Immunofluorescence

This process has been described in detail in our previous work [31]. IEC-6 cells were cultured in 6-well plates (4 × 10^5^ cells/well) and incubated for 24 h, and then stimulated with MOLP-L (25 µg/mL), MOLP-M (50 µg/mL), MOLP-H (100 µg/mL), or LPS (50 μg/mL) for 24 h, respectively. The dewaxed slices were put in water in a container filled with citrate buffer, boiled on high heat for about 5 min, then turned to medium heat and boiled for 15 min, a total of three times, and taken out each time to reduce evaporation loss. Isothermal distilled water was added to the liquid solution, and the container was taken out of the microwave and left at room temperature to cool slowly. After antigen retrieval, it was washed once with PBS for 5 min. The repaired slices were sealed with 3% bovine serum albumin (BSA) for 1 h. Then, the cells were placed in a wet box and incubated overnight at 4 °C with an appropriate amount of diluted primary antibody; after that, they were washed three times with PBS for 5 min each after being equilibrated at room temperature. The appropriate fluorescent secondary antibody was chosen and incubated in a wet box for 2 h in the dark, then washed three times with PBS for 10 min each. Then, it was stained with 4′,6-diamidino-2-phenylindole (DAPI) for 10 min and washed with PBS three times for 5 min each. Finally, the slides were fixed with an anti-fluorescence quencher and observed with a fluorescence microscope.

### 2.10. Statistical Analysis

The data are expressed as (mean ± SEM). To determine statistical differences among the groups, ANOVA was employed, followed by Duncan’s multiple comparison test. All statistical analyses were conducted utilising IBM-SPSS Statistics (Version 22.0.0.), and graphical representations were created using GraphPad-Prism software (Version 8.0.0.) Significance was considered at *p* < 0.05, *p* < 0.01, or *p* < 0.001.

## 3. Results and Discussion

### 3.1. Impact of MOLP on Cell Viability

**Figure 1:**
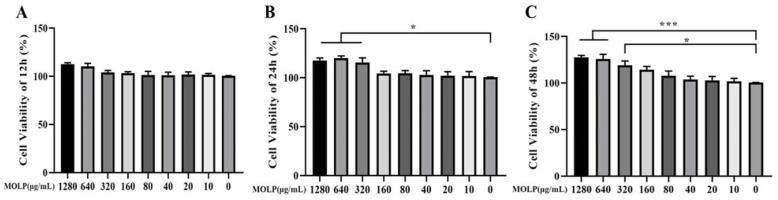
Impact of MOLP on IEC6 viability at (**A**) 12 h, (**B**) one day, and (**C**) two days. Results are shown as mean ± SEM (n = 3), with statistical significance denoted by * *p* < 0.05 and *** *p* < 0.001 compared to the Control group.

The influence of MOLP on cell viability was assessed using the CCK-8 assay. IEC6 cells were exposed to various concentrations of MOLP for a duration of 12, 24, and 48 h. As depicted in Figure 1A–C, in comparison to the control group, MOLP treatment did not adversely affect cell viability; in fact, it significantly enhanced (*p* < 0.05 and *p* < 0.001) cell viability at concentrations between 320 and 1280 µg/mL. For concentrations ranging from 10 to 160 µg/mL, the effect on cell proliferation was found to be concentration-dependent. Consequently, subsequent experiments were conducted with MOLP at concentrations of 25, 50, and 100 µg/mL.

### 3.2. Impact of MOLP on IEC-6 Cell Migration

**Figure 2:**
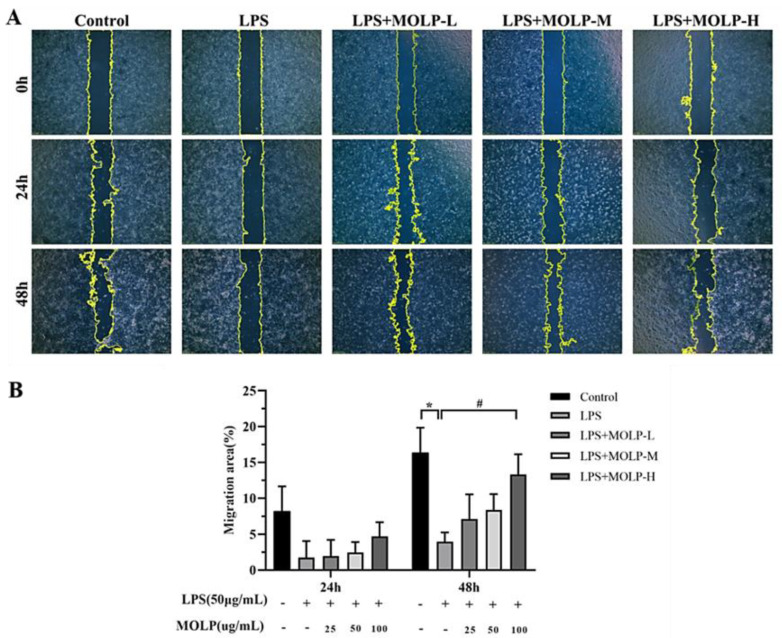
The impact of MOLP on the migration of IEC6 cells (**A**) was analysed statistically as presented (**B**). The results are shown as mean ± SEM (n = 3), with * *p* < 0.05 indicating significant differences between LPS and the Control, and # *p* < 0.05 indicating significance between the highest concentration of MOLP (MOLP-H) and LPS.

IEC6 cells were cultured to confluence in a 6-well plate and then subjected to repeated scraping. Subsequently, the cells were treated with LPS (50 µg/mL) and varying concentrations of MOLP (25, 50, and 100 µg/mL) for two days. LPS significantly reduced (*p* < 0.05) the migratory ability of IEC-6 cells at the 48 h time point compared to the Control group. However, at the highest concentration of MOLP, a significant increase (*p* < 0.05) in cell migration was observed in comparison to the LPS-treated group at the 48 h mark (Figure 2A,B).

Earlier research has shown that epithelial cell migration plays a crucial role in the healing of intestinal injuries. During this process, IECs migrate to the damaged site and rapidly reestablish the mucosal surface within a matter of hours [3,37]. Moreover, the proliferation of IECs has been shown to restore the integrity of the intestinal mucosa within one to two days [38]. L-lactate has been reported to facilitate the migration of IECs, thus helping to alleviate colitis over a one-day period [39]. Similarly, polysaccharides extracted from *si-jun-zi decoction* (SJZD) have been found to promote IEC-6 cell migration within 24 h [40], and interleukin-33, a cytokine similar to interleukin-1, has also been shown to enhance cell movement in IEC-6 cell lines [41].

### 3.3. MOLP Decreases Apoptosis in LPS-Activated IEC6 Cells

**Figure 3:**
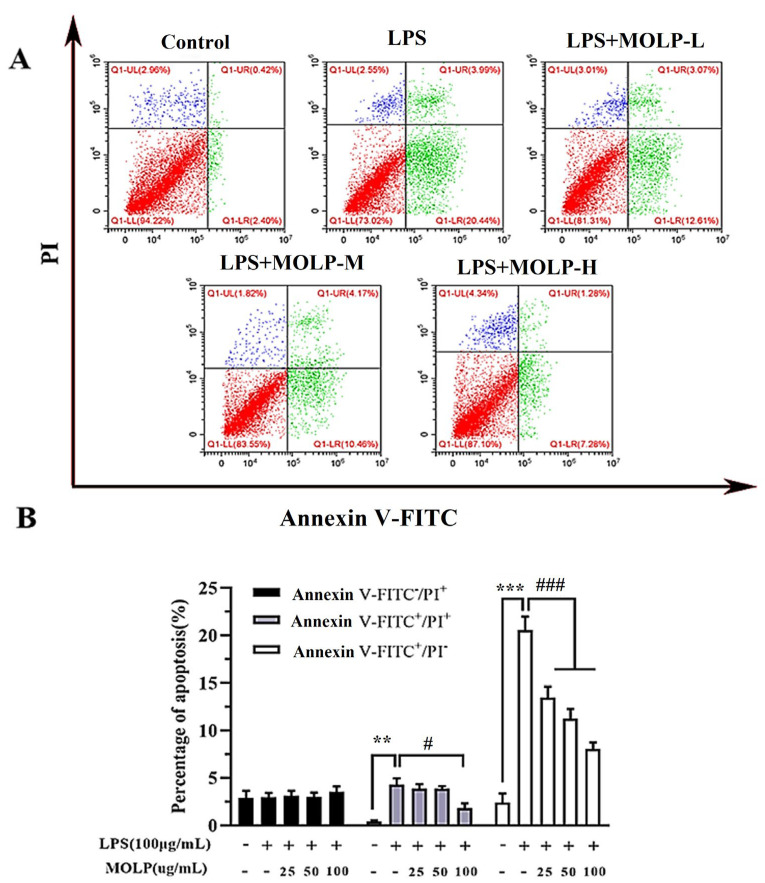
MOLP promotes apoptosis in LPS-stimulated IEC6 cells. The effect of MOLP on apoptosis induction in IEC6 cells following LPS stimulation was evaluated through flow cytometry (**A**) and statistically analysed (**B**). Cells were classified as viable apoptotic (Annexin: V^+^/PI^−^), non-viable apoptotic (Annexin: V^+^/PI^+^), and necrotic (Annexin V^−^/PI^+^). The results are displayed as mean ± SEM (n = 3). Significant differences are denoted as ** *p* < 0.01 and *** *p* < 0.001 between LPS and the Control; # *p* < 0.05 and ### *p* < 0.001 indicate significant differences between various MOLP concentrations (MOLP-L, MOLP-M, MOLP-H) and LPS.

LPS treatment resulted in a significant increase (*p* < 0.05) in the apoptosis rate of IEC6 cells when compared to the Control group. However, administration of all three doses of MOLP significantly reduced (*p* < 0.05) the incidence of cell apoptosis. These findings suggest that MOLP mitigates LPS-induced apoptosis in IEC-6 cellular models (Figure 3A,B). Similar to prior studies, it has been shown that *echinacoside* (ECH) effectively suppresses apoptosis in LPS-induced cells [36] and that *Ganoderma atrum* (*G. atrum*) polysaccharide (PSG-1) can inhibit apoptosis induced by acrolein [42]. These findings indicate that MOLP can inhibit LPS-induced apoptosis in IEC6 cells.

### 3.4. MOLP Suppresses Pro-Inflammatory Cytokine Production in LPS-Stimulated IEC6 Cells

**Figure 4:**
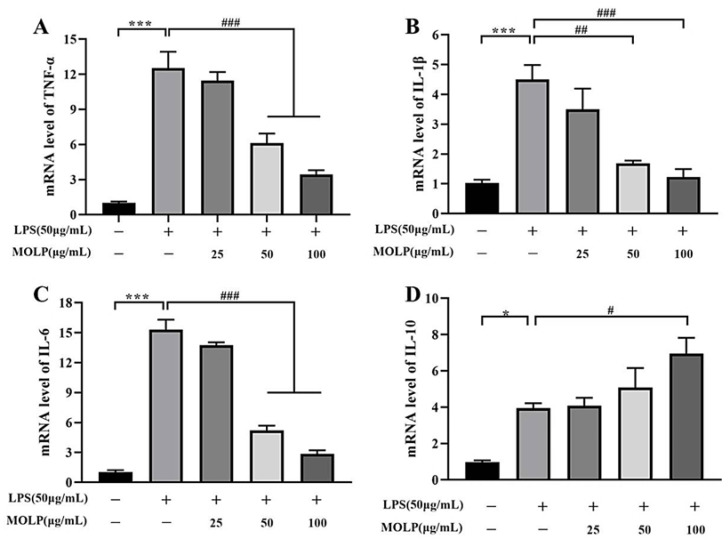
Impact of MOLP on mRNA expression levels of pro-inflammatory cytokines in LPS-activated IEC6 cells. The influence of MOLP on the mRNA expression of pro-inflammatory cytokines in IEC6 cells stimulated with LPS was examined. The cytokines analysed were (**A**) *TNF-α*, (**B**) *IL-1β*, (**C**) *IL-6,* and (**D**) *IL-10*. The data are presented as mean ± SEM (n = 3). Statistical significance is denoted as * *p* < 0.05 and *** *p* < 0.001 for comparisons between LPS and Control, and # *p* < 0.05, ## *p* < 0.01, ### *p* < 0.001 for comparisons between MOLP-L, -M, -H, and LPS.

To assess the anti-inflammatory properties of MOLP in LPS-activated IEC6 cells, cytokines mRNA expression levels were quantified utilising RT-qPCR. As illustrated in Figure 4, the LPS-treated model group showed a significant increase (*p* < 0.001) in mRNA expression levels of *TNF-α*, *IL-1β,* and *IL-6*, along with a notable decrease (*p* < 0.05) in *IL-10* levels when compared to the Control group. In contrast, treatment with medium and high concentrations of MOLP (MOLP-M and MOLP-H) significantly reduced (*p* < 0.001), the levels of *TNF-α*, *IL-1β,* and *IL-6* (Figure 4A–C). Notably, *IL-10* levels significantly increased (*p* < 0.05) in the MOLP-H treatment group (Figure 4D). These observations confirm that MOLP effectively suppresses the production of pro-inflammatory cytokines in LPS-stimulated IEC-6 cells. This aligns with previous research indicating that polysaccharides derived from *M. oleifera* roots (MRP-1) markedly reduced *TNF-α* mRNA expression [43]. Additionally, *Lactobacillus rhamnosus* CY12 strain (*L. rhamnosus* CY12) significantly downregulated the mRNA levels of *TNF-α*, *IL-1β,* and *IL-6* in Caco-2 cells [44], while ECH treatments decreased *TNF-α* and *IL-6* mRNA expression and increased *IL-10* and *TGF-β1* levels in LPS-stimulated IEC6 cells [36]. These findings illustrate the anti-inflammatory role of MOLP in reducing pro-inflammatory cytokine production in LPS-activated IEC6 cells.

### 3.5. MOLP Suppresses the TLR4/MyD88/NF-κB Signalling Pathway in LPS-Activated IEC6 Cells

The activation of NF-κB plays a significant role in regulating the production of pro-inflammatory cytokines [45]. Disruption of NF-κB signalling, whether through complete suppression or chronic activation, in IECs disturbs their balance and leads to intestinal inflammation. Therefore, maintaining NF-κB activation within an optimal range may help prevent intestinal inflammation [46].

**Figure 5:**
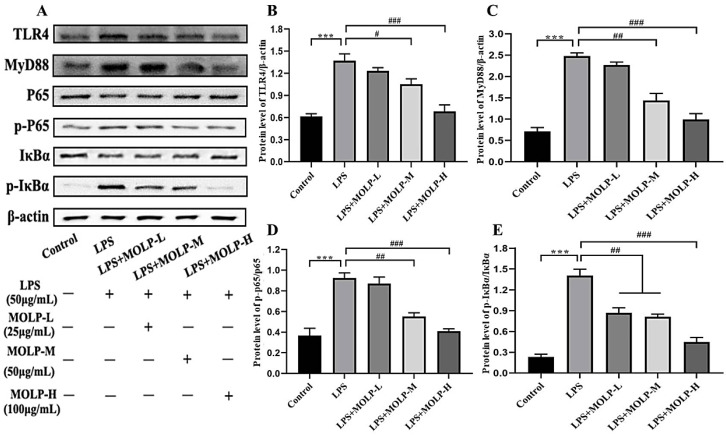
Impact of MOLP on inflammatory signalling pathways in IEC6 Cells. The influence of MOLP on inflammatory signalling pathways in IEC-6 cells was investigated. Key signalling proteins were evaluated utilising (**A**) Western blot analysis, with the relative protein levels quantified for (**B**) TLR4, (**C**) MyD88, NF-κB, (**D**) p65/p-P65, and (**E**) IκBα/p-IκBα; see Supplementary Files. The results are presented as mean ± SEM (n = 3). Statistical significance is denoted as *** *p* < 0.001 for comparisons between LPS and Control, and # *p* < 0.05, ## *p* < 0.01, ### *p* < 0.001 for comparisons among MOLP-L, -M, and -H against LPS.

As illustrated in Figure 5A–E, LPS significantly increased (*p* < 0.01) the protein levels of Toll-like receptor-4 (TLR4), myeloid differentiation primary response 88 (MyD88), phosphorylated P65 (p-P65), and phosphorylated IκB-α (p-IκB-α) in comparison to the Control group, suggesting that LPS may activate the NF-κB signalling pathway via the phosphorylation of P65 and IκB-α. Conversely, all three doses of MOLP significantly reduced (*p* < 0.05) the protein levels of MyD88, TLR4, p-P65, and p-IκB-α in IEC6 cells.

This observation aligns with previous findings showing that the *L. rhamnosus* CY12 strain inhibited the activation of the TLR4 and NF-κB signalling pathways in LPS-stimulated Caco-2 cells [44]. These results suggest that MOLP suppresses inflammatory signalling pathways in LPS-activated IEC-6 cells.

### 3.6. MOLP Protects Tight Junction Proteins in LPS-Stimulated IEC6 Cells

**Figure 6:**
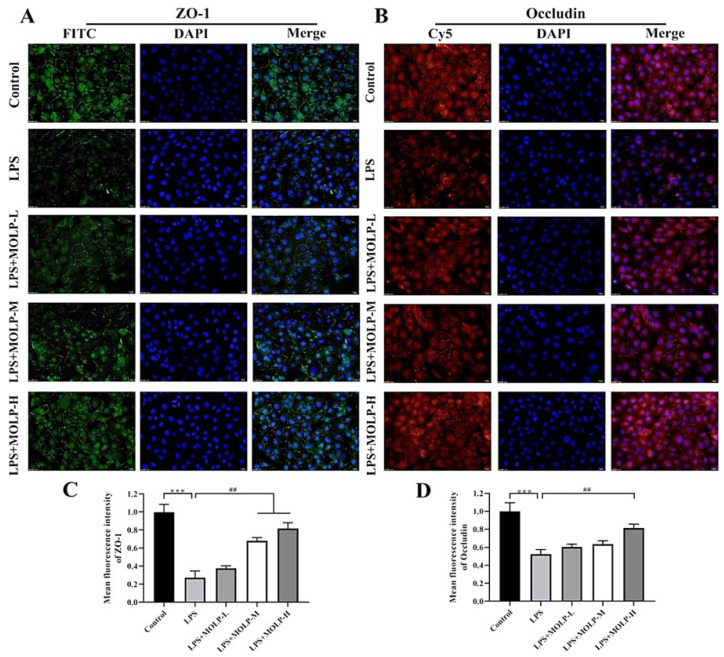
Effect of MOLP on the immunolocalisation and relative fluorescence intensity of TJ proteins in LPS-stimulated IEC6 cells. Panels (**A**,**B**) show the immunolocalisation of ZO-1 and occludin, respectively, in LPS-stimulated IEC-6 cells. Panels (**C**,**D**) present the quantitative results derived from (**A**,**B**). The images were captured at 100× magnification, with a scale bar representing 50 μm. Results are shown as mean ± SEM (n = 3), with *** *p* < 0.001 indicating significance between LPS and Control, and ## *p* < 0.01 between MOLP-M, MOLP-H, and LPS.

These findings indicate that MOLP has a suppressive effect on inflammatory signalling pathways in LPS-activated IEC6 cells [47,48,49]. Figure 6A,B represent the expression of occludin, and the fluorescence intensity of ZO-1 was notably diminished in the LPS-treated group. However, treatment with medium and high concentrations of MOLP (M and H) maintained the expression and localisation of these tight junction proteins. This suggests that MOLP's protective effects on the epithelial barrier may stem from its ability to restore compromised tight junctions in IEC-6 cells exposed to LPS.

The present findings are in line with previous investigations, which demonstrated that the *L. rhamnosus* CY12 strain boosted the levels of tight junction proteins by up-regulating the expression of ZO-1 and occludin in Caco-2 cells [44]. Similarly, PSG-1 treatment increased the levels of ZO-1 and occludin in acrolein-induced IEC6 cells [42]. These results imply that MOLP supplementation could be advantageous for maintaining the expression of tight junction (TJ) proteins, thereby preserving the integrity of the epithelial barrier.

Briefly, the present study elucidates the beneficial impact of MOLP on the viability, migration, and apoptosis of IEC-6 cells, particularly under LPS-induced stress conditions. The results indicate that MOLP significantly promotes cell proliferation at optimal concentrations and effectively mitigates LPS-induced inhibition of cell migration. Furthermore, the polysaccharides demonstrated a pronounced ability to reduce apoptosis rates, thereby safeguarding cell integrity. These findings suggest that MOLP plays a vital role in maintaining cellular homeostasis and could be instrumental in accelerating the repair processes of intestinal epithelial cells following injury or inflammation.

Additionally, this study highlights the potent anti-inflammatory properties of MOLP, as evidenced by the substantial reduction in pro-inflammatory cytokine levels and the suppression of the TLR4/MyD88/NF-κB signalling pathway in LPS-activated IEC6 cells. This anti-inflammatory effect aligns with previous research, underscoring MOLP's potential as a therapeutic agent in managing intestinal inflammation. By down-regulating key pro-inflammatory cytokines and signalling pathways, MOLP not only alleviates inflammation but also supports the overall functional stability of the intestinal mucosa. This multifaceted anti-inflammatory action positions MOLP as a promising candidate for further exploration in the context of inflammatory bowel diseases and other gastrointestinal disorders marked by chronic inflammation. In this aspect, Wang et al. [50] found that the administration of MOLP significantly altered the tumour microenvironment, promoting the shift of tumour-associated macrophages from an immunosuppressive M2 phenotype to an anti-tumour M1 phenotype. On the other side, Husien et al. [51] assessed the impact of MOLP on gut microbiota in mice with DSS-induced ulcerative colitis (UC). Mice were treated with DSS to induce colitis and then administered a high dose of MOLP (100 mg/kg/day). Fecal samples were analysed using 16S rDNA high-throughput sequencing. The results showed that MOLP treatment increased beneficial bacteria, such as *Firmicutes*, and decreased harmful bacteria like Helicobacter in the DSS-induced UC mice. These findings suggest that MOLP could act as a prebiotic, offering potential for inclusion in dietary supplements or pharmaceutical applications to improve gut health and manage ulcerative colitis.

The present study highlights the potential therapeutic benefits of Moringa Oleifera Leaf Powder (MOLP) for enhancing intestinal health, particularly in the context of LPS-activated IEC6 cells. Our findings regarding the potential therapeutic benefits of MOLP align with previous research conducted by Camilleri et al. [52], Arora and Arora [53], Srivastava et al. [54], and Abd El-Hack et al. [55], which also demonstrates the beneficial effects of *M. oleifera*. Similarly, MOLP exhibited significant anti-inflammatory properties by reducing the levels of pro-inflammatory cytokines such as *TNF-α*, *IL-1β,* and *IL-6*. This reduction was achieved by inhibiting key inflammatory signalling pathways, including TLR-4, MyD88, pIκB-α, and phosphorylated NF-κB p65. Moreover, MOLP significantly promoted cell migration and viability, further elucidating its role in fostering an optimal cellular environment for intestinal health.

A notable aspect of MOLP functionality is its impact on TJ proteins. A previous study by Shen et al. [56] highlighted the importance of TJ proteins, such as ZO-1 and occludin, in maintaining epithelial barrier integrity. This study demonstrates that MOLP restores the expression of these TJ proteins, which had been disrupted by LPS, corroborating findings from other studies that emphasise the role of TJ integrity in gastrointestinal health. Additionally, the examination of MOLP in this study echoes the findings of Liu et al. [57], who reported on MOLP’s ability to influence intestinal permeability through the modulation of tight junctions. The anti-inflammatory and antioxidant properties observed in both in vitro and in vivo settings suggest that MOLP holds substantial promise for clinical applications aimed at preventing or treating intestinal disorders, particularly those stemming from inflammation and oxidative stress. The findings of this research are consistent with those of Gu et al. [58], who demonstrated that polysaccharides from MOLP not only possess antioxidative properties but also aid in glycemic control, further broadening the potential therapeutic applications of Moringa-derived bioactive compounds.

Furthermore, this study adds to the growing body of evidence supporting the development of natural therapeutic agents in veterinary and human medicine. In this regard, Paradowska et al. [59] underscored the potential for bioactive compounds to replace traditional antimicrobial agents in poultry farming, providing a compelling argument for the inclusion of MOLP as a functional additive in animal feed. This aligns with our findings that MOLP enhances intestinal health and exemplifies the broad applicability of MOLP in both agricultural and clinical settings.

Additionally, several reliable studies demonstrate the diverse potential of MOLP across different animal models and conditions. Zvinorova et al. [60] highlight the adaptative growth features in nutrient-restricted environments in weanling Sprague Dawley rats while mitigating severe effects on liver or GIT function. Khalid et al. [61] provide insights into the application of MOLP to mitigate heat stress in New Zealand White rabbits, particularly focusing on enhancing gut integrity and reducing inflammation. Khan et al. [62] underscore the potential of MOLP as a natural alternative to subtherapeutic antibiotics in broiler chickens, effectively improving intestinal structure and mucin production. Husien et al. [31,51] expand MOLP’s therapeutic horizon to ulcerative colitis, showing its ability to counteract inflammation and support beneficial intestinal flora in mouse models. Collectively, these studies suggest that MOLP could be instrumental in improving animal health through its nutritional and therapeutic properties, offering avenues for enhancing growth performance and managing specific health conditions via dietary supplementation.

Lastly, the potential of MOLP to preserve and restore the expression of TJ proteins, such as ZO-1 and occludin, reinforces its role in enhancing the epithelial barrier function. The maintenance of TJ integrity is crucial for preventing barrier permeability, which is often compromised in various intestinal pathologies. By stabilising these junctions, MOLP helps maintain the selective permeability that is essential for the proper function of the gastrointestinal tract. This aspect, coupled with MOLP antioxidant properties, underscores the comprehensive protective effect of MOLP on maintaining intestinal health. Future investigations could delve deeper into the molecular mechanisms governing these benefits, providing a robust foundation for the clinical application of MOLP in gastrointestinal health and disease prevention.

Future research should aim to elucidate the precise molecular mechanisms by which MOLP exerts its effects. Studies could investigate the synergistic interactions between MOLP and other bioactive compounds to develop comprehensive strategies for maintaining gut health and preventing disease. Given the increasing interest in natural and plant-derived compounds as alternatives to synthetic drugs, further exploration into the clinical applications of MOLP could pave the way for innovative dietary supplements and pharmaceutical interventions designed to enhance intestinal health and mitigate related disorders.

## 4. Conclusions

This study demonstrated that polysaccharides extracted from MOL have a significant impact on the migratory and proliferative abilities of IEC-6 cells in vitro. Furthermore, these polysaccharides enhance the integrity of the epithelial barrier by increasing the expression levels of TJ proteins. These findings indicate that MOLP could offer promising therapeutic benefits, potentially aiding in the treatment of intestinal disorders related to tissue damage and supporting overall intestinal mucosal health. In addition to their beneficial effects on epithelial cells, MOLP might also possess anti-inflammatory and antioxidant properties that contribute to their protective role in gastrointestinal health.

## Figures and Tables

**Table 1:** Genes and sequences utilised for RT-qPCR in the current study.

Gene		Sequence (5′-----3′)
*TNFα*	**F1-**	TGAAGCAGCAGCCAGCAA
	**R1-**	GCAGCCTGTCTCCTTCTATGA
*IL1β*	**F2-**	CCAGCAGGTTATCATCACATCC
	**R2-**	ATCTCGCAGCAGCACATCA
*IL6*	**F3-**	AATTAAGCCTCCGACTTGTGAA
	**R3-**	TTCCATCCAGTTGCCTTCTTG
*IL10*	**F4-**	GGCAGCCTTGTCCCTTG
	**R4-**	AACATACTGCTAACCGACTCCTT
*GAPDH*	**F5-**	CACCATCTTCCAGGAGCGAG
	**R5-**	GGGGCCATCCACAGTCTTC

## Data Availability

Information is presented in the manuscript or the accompanying Supplementary Files.

## Supplementary Materials

The following supporting information can be downloaded at: https://www.mdpi.com/article/10.3390/ani14233508/s1, All data generated or analyzed during this study are included in this manuscript.
