# Supplementary material for: The Anti-Inflammatory Properties of Polysaccharides Extracted from Moringa oleifera Leaves on IEC6 Cells Stimulated with Lipopolysaccharide In Vitro
Source: Animals (Basel). 2024 Dec 4;14(23):3508. doi: 10.3390/ani14233508 (PMC11640475; doi:10.3390/ani14233508)
Supplement: Supplementary file 1 [file animals-14-03508-s001.zip › animals-3251614-supplementary.pdf]

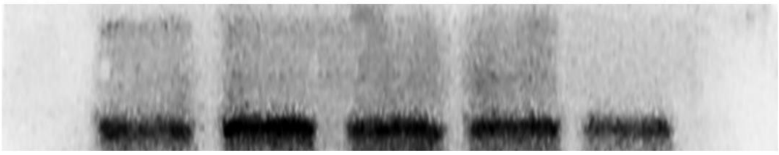

Figure S1. Western blot analysis, with the relative protein levels quantified for *TLR4*, Notably, the screening for taking the blots were taken directly as it's via the documentation system.

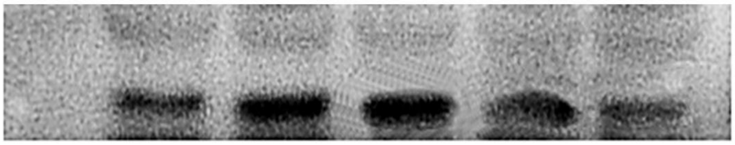

Figure S2. Western blot analysis, with the relative protein levels quantified for *MyD88* Notably, the screening for taking the blots were taken directly as it's via the documentation system.

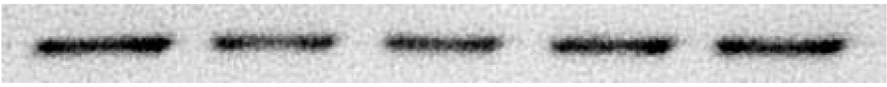

Figure S3. Western blot analysis, with the relative protein levels quantified for *P65.* Notably, the screening for taking the blots were taken directly as it's via the documentation system.

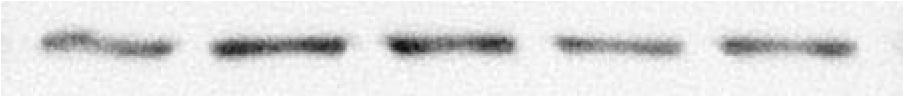

Figure S4. Western blot analysis, with the relative protein levels quantified for *p-P65.* Notably, the screening for taking the blots were taken directly as it's via the documentation system.

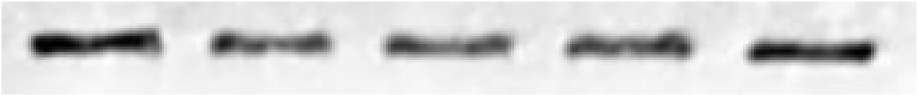

Figure S5. Western blot analysis, with the relative protein levels quantified for *IκBα.* Notably, the screening for taking the blots were taken directly as it's via the documentation system.

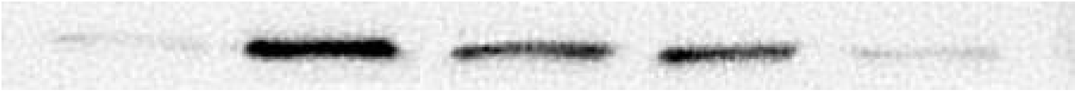

Figure S6. Western blot analysis, with the relative protein levels quantified for *p-IκBα.* Notably, the screening for taking the blots were taken directly as it's via the documentation system.

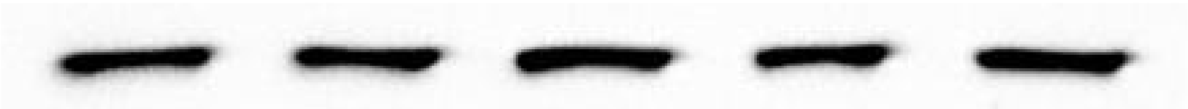

Figure S7. Western blot analysis, with the relative protein levels quantified for *B-Actin.* Notably, the screening for taking the blots were taken directly as it's via the documentation system.
